# The Novel Anaerobiosis-Responsive Overlapping Gene *ano* Is Overlapping Antisense to the Annotated Gene ECs2385 of *Escherichia coli* O157:H7 Sakai

**DOI:** 10.3389/fmicb.2018.00931

**Published:** 2018-05-14

**Authors:** Sarah M. Hücker, Sonja Vanderhaeghen, Isabel Abellan-Schneyder, Siegfried Scherer, Klaus Neuhaus

**Affiliations:** ^1^Chair for Microbial Ecology, Technical University of Munich, Freising, Germany; ^2^Institute for Food & Health, Technical University of Munich, Freising, Germany; ^3^Core Facility Microbiome/NGS, Institute for Food & Health, Technical University of Munich, Freising, Germany

**Keywords:** anaerobiosis, overlapping gene, EHEC O157:H7, RNAseq, ribosomal footprinting, *ano*

## Abstract

Current notion presumes that only one protein is encoded at a given bacterial genetic locus. However, transcription and translation of an overlapping open reading frame (ORF) of 186 bp length were discovered by RNAseq and RIBOseq experiments. This ORF is almost completely embedded in the annotated L,D-transpeptidase gene ECs2385 of *Escherichia coli* O157:H7 Sakai in the antisense reading frame -3. The ORF is transcribed as part of a bicistronic mRNA, which includes the annotated upstream gene ECs2384, encoding a murein lipoprotein. The transcriptional start site of the operon resides 38 bp upstream of the ECs2384 start codon and is driven by a predicted σ^70^ promoter, which is constitutively active under different growth conditions. The bicistronic operon contains a ρ-independent terminator just upstream of the novel gene, significantly decreasing its transcription. The novel gene can be stably expressed as an EGFP-fusion protein and a translationally arrested mutant of *ano*, unable to produce the protein, shows a growth advantage in competitive growth experiments compared to the wild type under anaerobiosis. Therefore, the novel antisense overlapping gene is named *ano* (anaerobiosis responsive overlapping gene). A phylostratigraphic analysis indicates that *ano* originated very recently *de novo* by overprinting after the *Escherichia/Shigella* clade separated from other enterobacteria. Therefore, *ano* is one of the very rare cases of overlapping genes known in the genus *Escherichia*.

## Introduction

*Escherichia coli* strains are classified as enterohemorrhagic *E. coli* (EHEC) when they possess Shiga-toxin genes and the locus of enterocyte effacement (Sadiq et al., 2014). The EHEC strain O157:H7 Sakai was isolated from an outbreak in Japan in 1996. It has a genome of 5.5 Mb (Hayashi et al., 2001), which is 20% larger than the genome of *E. coli* K12, probably due to DNA acquired by horizontal gene transfer and integration of 24 prophages (Sadiq et al., 2014). In humans, EHEC causes hemorrhagic colitis and the disease can progress to the life-threatening hemolytic uremic syndrome (Lim et al., 2010). Neither targeted therapy nor vaccination is available, and antibiotics even promote a fatal outcome by Shiga-toxin induction (Wong et al., 2000). The serotype O157:H7 is the most frequent clinical isolate causing 100,000 reported infections per year in United States (Eppinger and Cebula, 2015). Transmission mainly occurs via consumption of contaminated food, e.g., undercooked beef or fresh produce, but also person-to-person and animal-to-person spread is possible (Lim et al., 2010).

Enterohemorrhagic *E. coli* thrives in many environmental niches: while the major reservoir are cattle, EHEC also colonizes other mammals, birds, fish, insects (Persad and LeJeune, 2014), and the protozoan *Acanthamoeba polyphaga* (Barker et al., 1999). Insects can serve as transmission vectors (Semenov et al., 2010; Wasala et al., 2013). Another important reservoir are green leaf plants, where EHEC colonizes the stomata (Saldana et al., 2011) and roots (Hou et al., 2013), and is even internalized in seedlings (Jayaraman et al., 2014). In addition, EHEC persists for several weeks in beforehand sterilized soil and water at cold temperature (Duffitt et al., 2011). Cycling between different hosts and various biotic and abiotic habitats occurs frequently. These different life styles display variable challenges and require expression of changing sets of genes.

Therefore, Next Generation Sequencing has been used as a powerful tool to investigate global gene expression at different levels in EHEC. Strand-specific RNAseq allows the quantification of transcription (Flaherty et al., 2011). For example, the transcriptome of EHEC strain EDL933 was determined under 11 different growth conditions (i.e., radish sprouts, cow dung, antibiotic treatment, etc.) and shows differential expression of many genes (Landstorfer et al., 2014). Besides signals mapping to annotated genes, RNAseq experiments resulted in many reads mapping to intergenic regions or antisense to annotated genes (Dornenburg et al., 2010). In the past, those signals were interpreted to represent ncRNA (Lasa et al., 2011; Raghavan et al., 2011) or just pervasive transcription (Wade and Grainger, 2014). Today, RIBOseq allows investigation of the global translatome (Ingolia et al., 2009) by sequencing only mRNA, which is protected by ribosomes. When RIBOseq and RNAseq are combined, the translatability of a certain open reading frame (ORF) can be determined and ncRNA can be distinguished from protein coding mRNA (Neuhaus et al., 2017). Indeed, many RNAseq signals outside of annotated genes also show RIBOseq signals, leading to the discovery of hundreds of translated ORFs in eukaryotes (Aspden et al., 2014; Bazzini et al., 2014; Ingolia et al., 2014; Ruiz-Orera et al., 2014; Ji et al., 2015; Calviello et al., 2016). Combined RNAseq and RIBOseq also detected 130 novel genes in *Salmonella enterica* Typhimurium (Baek et al., 2017), 72 novel genes in the intergenic regions of EHEC EDL933 (Neuhaus et al., 2016), and 465 novel genes in EHEC Sakai (Hücker et al., 2017). However, functional characterization of those translated ORFs is largely lacking.

Overlapping genes (OLGs) are encoded by a different reading frame within an annotated protein-coding gene. While OLGs have been described for viruses (Chirico et al., 2010; Torres et al., 2013; Fernandes et al., 2016; Taylor and Strebel, 2017), they have only very rarely been reported in prokaryotes. The notion of “one gene – one enzyme” and, as an implicit consequence, “one locus – one gene” has survived as a general mindset until today (Beadle and Tatum, 1941). In EHEC, just three antisense overlapping gene pairs have been characterized so far: *htgA/yaaW* (Delaye et al., 2008; Fellner et al., 2014), *nog1/citC* (Fellner et al., 2015), and *laoB*/ECs5115 (Hücker et al., 2018). Only five additional OLG pairs are known from other *E. coli* strains: *yghW/morA* (Kurata et al., 2013), *pic/setB* (Behrens et al., 2002), *ardD/tniA* (Balabanov et al., 2012), *aatS/aatC* (Haycocks and Grainger, 2016), and *tnpA/astA* (McVeigh et al., 2000). Here, we report experimental evidence for a fourth OLG pair in EHEC: the novel gene *ano* overlaps in antisense to the annotated L,D-transpeptidase gene ECs2385 and encodes a functional, stress-related protein.

## Materials and Methods

Bacterial strains and plasmids used in this study are listed in **Supplementary Table S1**. Oligonucleotides used are listed in **Supplementary Table S2**.

### Determination of Transcriptional Start Site by 5′ RACE

The total RNA of an overnight culture in LB medium of *E. coli* O157:H7 Sakai (GenBank Accession No. NC_002695) (Hayashi et al., 2001) was isolated with Trizol (Thermo Fisher Scientific). The kit 5′ RACE System for Rapid Amplification of cDNA Ends, Version 2.0 (Invitrogen) was used according to the manual using the primer *ano*+159R for reverse transcription, *ano*+81R for the first PCR and *ano*+56R for the second. After the second PCR, the dominant product was excised from the agarose gel and purified with the GenElute^TM^ Gel Extraction Kit (Sigma-Aldrich). The PCR product was Sanger sequenced by Eurofins with oligonucleotide *ano*+56R.

### Reverse Transcription Polymerase Chain Reaction (RT-PCR)

Total RNA of 500 μl overnight EHEC culture in LB medium was isolated with Trizol. Remaining DNA was digested using 1 μl of 2 U/μl TURBO^TM^ DNase (Thermo Fisher Scientific) for 1 h at 37°C. After RNA purification by ethanol precipitation, reverse transcription with 500 ng RNA as template was performed using 200 U SuperScript^TM^ III Reverse Transcriptase (Thermo Fisher Scientific) according to the manual. The obtained cDNA was used as template for a PCR with a primer pair (ECs2384+4F and *ano*+124R) spanning ECs2384 and *ano*.

### Quantitative Reverse Transcription Polymerase Chain Reaction (qRT-PCR)

Relative quantification of ECs2384 and *ano* mRNA was performed at the following conditions: 0.5×LB at 37°C aerobically, OD_600_ value of 0.5 and 0.5×LB at 37°C anaerobically, OD_600_ of 0.5. RNA of 2 ml EHEC culture was isolated using the RNeasy Mini Kit (Qiagen). Cell lysis was performed using 200 μl lysozyme (15 mg/ml) in TE buffer at pH 8. Then, 15 μl proteinase K (20 mg/ml) were added and the sample was incubated for 15 min at room temperature. The following steps were performed according to the manual, except the on-column DNase digestion was skipped and instead 10 μg RNA were incubated with 2 U TURBO^TM^ DNase (Thermo Fisher Scientific) for 1 h at 37°C. After RNA purification by ethanol precipitation, reverse transcription with 2 μg RNA as template was performed using 200 U SuperScript^TM^ III Reverse Transcriptase (Thermo Fisher Scientific) and a Random Nonamer primer (GE Healthcare) according to the manual. One μl cDNA was used as template for the quantitative reverse transcription polymerase chain reaction (qRT-PCR) with the SYBR^®^ Select Master Mix (Applied Biosystems) on a CFX96^TM^ Real-Time machine (Bio-Rad). The ΔΔCt method was used for quantification (Pfaffl, 2001) and 16S rRNA was used as reference. Primers used included rrsHR and rrsHF for the 16S rRNA gene, *ano*+14F and *ano*+161R for the *ano* gene, and ECs2384+4F and ECs2384+215R for ECs2384.

### Cloning of pProbe-NT EGFP Reporter Plasmid and Determination of Promoter Activity

The genomic region 300 bp upstream of the determined transcriptional start site (TSS) of *ano* was amplified by PCR (primers ECs2384-*Sal*I-320F and ECs2384-*EcoR*I-40R) and restriction enzyme cut sites for *Sal*I and *EcoR*I were introduced. The PCR product was cloned into the plasmid pProbe-NT (Miller et al., 2000) and transformed into *E. coli* Top10. The plasmid sequence was verified by Sanger sequencing (Eurofins). Overnight cultures of *E. coli* Top10 + pProbe-NT and *E. coli* Top10 + pProbe-NT-PromoterTSS were used for 1:100 inoculation of 10 ml 0.5×LB medium with 30 μg/ml kanamycin. Growth in 0.5×LB was investigated for promoter activity using the following conditions: plain medium, at pH 5, at pH 8.2, plus 400 mM NaCl, plus 0.5 mM CuCl_2_, plus 2 mM formic acid or plus 2.5 mM malonic acid. Cultures were incubated at 37°C and 150 rpm until an OD_600_ of 0.5 was reached (3 to 8 h, dependent on growth condition). Next, the cells were pelleted, washed once with phosphate-buffered saline (PBS) and resuspended in 1 ml PBS. The OD_600_ was adjusted to 0.3 and 0.6. Four times each 200 μl bacterial suspension were pipetted in a black microtiter plate and the fluorescence was measured (Wallac Victor^3^, Perkin Elmer Life Science, excitation 485 nm, emission 535 nm, measuring time 1 s). The fluorescence of *E. coli* Top10 without plasmid was subtracted as background. Promoter activity at anaerobic conditions was determined with the following changes of the protocol: 15 ml 0.5×LB with 30 μg/ml kanamycin (investigated conditions see above) inoculated 1:100 with overnight cultures of *E. coli* Top10 + pProbe-NT or *E. coli* Top10 + pProbe-NT-PromoterTSS in tightly closed 15 ml falcon tubes were incubated at 37°C and 150 rpm until an OD_600_ of 0.5 was reached (6–12 h dependent on growth condition). Subsequently, the cultures were transferred into Schott flasks and incubated for 15 min aerobically at 37°C and 150 rpm to allow the EGFP to mature. Cell harvest and measurement of fluorescence intensity was performed as described above. The experiment was performed in triplicate. Significance of changes was calculated by two-tailed Student’s *t*-test.

### Cloning of C-Terminal Ano-EGFP Fusion Proteins and Overexpression of Ano Protein

The *ano* sequence without the stop codon was amplified by PCR and restriction enzyme cut sites for *Pst*I and *Nco*I were introduced (primers ano_S1-*Pst*I-116F or ano_S2-*Pst*I-107F or *ano*_S3-PstI-95F or *ano*_S5-PstI+1F and *ano*-*Nco*I+167R; primer ECs2385-*Pst*I+1F and ECs2385-*NcoI*+983R). The PCR product was cloned into the plasmid pEGFP and transformed into *E. coli* Top10. Because the correct start codon of *ano* is unknown, pEGFP plasmids for the other possible start codons 1 (CTG), 2 (ATG), 3 (GTG), and 5 (CTG) were constructed (**Figure 2**). Cloning of an EGFP fusion protein for the fourth possible start codon (GTG) failed. As negative controls, also C-terminal EGFP fusion plasmids with translationally arrested *ano* sequences for every possible start codon were cloned (see below). The plasmid sequences were verified by Sanger sequencing (Eurofins). For fusion-protein overexpression, overnight cultures of *E. coli* Top10 + pEGFP, *E. coli* Top10 + pEGFP-*ano*_start1-5 and *E. coli* Top10 + pEGFP-*ano^∗^*_start1-5 were inoculated 1:100 in 10 ml 0.5×LB medium with 120 μg/ml ampicillin in duplicates. Cultures were incubated at 37°C and 150 rpm until an OD_600_ of 0.3 was reached. In one culture each, protein expression was induced by 10 mM IPTG. Incubation of induced and uninduced cultures was continued for 1 h and then cells were pelleted. The cells were washed once with PBS and the pellet was resuspended in 1 ml PBS. The OD_600_ was adjusted to 0.3 and 0.6. Four-times each 200 μl diluted culture were pipetted in a black microtiter plate and the fluorescence was measured (Wallac Victor^3^, Perkin Elmer Life Science, excitation 485 nm, emission 535 nm, measuring time 1 s). The fluorescence of *E. coli* Top10 without plasmid was subtracted as background. The experiment was performed in triplicate. Significance of changes was calculated by two-tailed Student’s *t*-test.

### Cloning of a Translationally Arrested *ano* Mutant

For cloning of the genomic translationally arrested mutant *ano^∗^*, the genome editing method of Kim et al. (2014) was adopted. The pHA_1887_ fragment and the selection cassette were amplified by PCR from the plasmid pTS2Cb (either primers pHA5F and pHA3R, or SM5F and SM3R). A point mutation leading to a premature stop codon was introduced into the *ano* sequence by PCR with the oligonucleotides HA3*ano*-115F and SM5*ano*mut+19R (3′ mutation fragment), and SM3*ano*mut-5F and HA5*ano*+174R (5′ mutation fragment). Because the plasmid pTS2Cb-*ano^∗^* was constructed by Gibson Assembly, the four PCR fragments had to contain overlapping sequences. In a total reaction volume of 20 μl, 200 fmol of each PCR fragment and the NEBuilder^®^ HiFi DNA Assembly Master Mix (NEB) were incubated at 50°C for 4 h. Two μl of the reaction were transformed into *E. coli* Top10 and plated on LB agar with 120 μg/ml ampicillin and 20 μg/ml chloramphenicol. Next, the mutation cassette was amplified by PCR using pTS2Cb-*ano^∗^* as template and primers HA3F and HA5R. The PCR product of correct size was purified from an agarose gel (GenElute^TM^ Gel Extraction Kit; Sigma-Aldrich). *E. coli* O157:H7 Sakai (Hayashi et al., 2001) transformed with the plasmid pSLTS were subsequently transformed with 75 ng of the mutation cassette. After incubation for 3 h at 30°C and 150 rpm in SOC medium, cultures were plated on LB agar plates with 120 μg/ml ampicillin and 20 μg/ml chloramphenicol and incubated at 30°C. One colony per plate was suspended in PBS. One-hundred μl of a 1:10 dilution with PBS were plated on LB agar with 100 μg/ml ampicillin and 100 ng/ml anhydrotetracycline for I-*Sce*I induction and incubated at 30°C overnight. Several colonies were streaked on plain LB agar and LB agar with 20 μg/ml chloramphenicol and incubated at 37°C overnight. Colonies, which were only able to grow on LB, were selected and the genomic area surrounding the mutation was amplified by PCR. In addition to the premature stop codon introduced, the restriction enzyme cut site for *Hga*I had been deleted, which enabled checking the PCR-product using *Hga*I. Introduction of the point mutation was confirmed by Sanger sequencing (Eurofins) for *Hga*I-negative PCR products.

### Competitive Growth Assays

Overnight cultures of EHEC Sakai wild type and EHEC Sakai *ano^∗^* were adjusted to an OD_600_ of 1.0 and then mixed in equal quantities (500 μl wild type + 500 μl mutant). Five-hundred μl of the mixture were pelleted and the cells were snap frozen in liquid nitrogen (control *t* = 0). Ten ml 0.5×LB medium were inoculated 1:3000 with the mixed EHEC culture and shaken at 37°C until the stationary growth phase was reached. The following conditions were investigated under aerobic conditions in 0.5×LB: plain medium, at pH 5.2, at pH 8, plus 400 mM NaCl, plus 0.5 mM CuCl_2_, plus 2 mM formic acid or plus 2.5 mM malonic acid. Additionally, the experiment was carried out anaerobically with the same supplements as described above using 15 ml 0.5×LB medium in tightly closed 15-ml falcon tubes. Cultures were incubated for 18 h at 37°C and 150 rpm. Next, 500 μl of culture were pelleted, 100 μl ddH_2_O were added, and the sample was heated to 95°C for 10 min. The crude DNA-preparation was used as template for a PCR with the primer pair *ano*-78F and *ano*+124R. The PCR product was Sanger sequenced (Eurofins) and the ratio between wild type and mutant was determined by comparing peak heights. The absolute numbers were transformed into percentage values of each condition and the values were normalized to a *t* = 0 ratio of 50:50 wild type to mutant. The experiment was performed in biological triplicates. Significance of changes was calculated by two-tailed Student’s *t*-test.

### Complementation of EHEC *ano^∗^*

To compensate for the *ano* genomic translational arrest, the intact *ano* ORF (start codon 2, ATG) was complemented on the plasmid pBAD/*Myc*-*His*-C *in trans*. In addition, plasmids of shorter *ano* sequences using the alternative start codons 4 (GTG) and 5 (CTG) were cloned likewise. First, the sequence of *ano* was amplified by PCR and restriction enzyme cut sites for *Nco*I and *Hind*III were introduced (primers *ano*_S2-*Nco*I-107F or *ano*_S4-*Nco*I-35F or *ano*_S5-*Nco*I+1F and *ano*-*Hind*III+170R). The PCR product was cloned into the plasmid pBAD/*Myc*-*His*-C and the plasmid was transformed into *E. coli* O157:H7 Sakai *ano^∗^*. As a negative control, similar plasmids containing the mutated *ano* ORF (*ano^∗^*) were cloned. Next, competitive growth experiments for the three possible start codons were performed, as described above, using *E. coli* O157:H7 Sakai *ano^∗^* + pBAD-*ano*-Start2/4/5 (complementation) and *E. coli* O157:H7 Sakai *ano^∗^* + pBAD-*ano^∗^*-Start2/4/5. Overnight cultures were supplemented with 120 μg/ml ampicillin, adjusted to an OD_600_ value of 1, mixed in equal ratio and inoculated into 15 ml 0.5×LB in tightly closed 15-ml falcon tubes in duplicates. One culture each was induced with 0.002% arabinose, the other left uninduced. After incubation at 37°C and 150 rpm for 18 h, plasmids were isolated using GenElute^TM^Plasmid Miniprep Kit (Sigma-Aldrich). With 20 ng isolated plasmids, a PCR was performed using oligonucleotides pBAD+208F and *ano*+124R. The PCR products were Sanger sequenced (Eurofins) and the ratio of intact *ano* over translationally arrested *ano* was determined in percent. The experiment was performed in biological triplicates. Significant changes were calculated by two-tailed Student’s *t*-test.

### Transcriptome and Translatome Sequencing

Strand-specific RNAseq and RIBOseq data (Hücker et al., 2017) were explored with regard to translated ORFs in antisense to annotated genes. Briefly, the bacteria had been grown at the following growth conditions: LB medium at 37°C, harvested at OD_600_ of 0.4, BHI medium at 37°C, harvested at OD_600_ of 0.1, and BHI medium supplemented with 4% NaCl at 14°C, harvested at OD_600_ of 0.1. LB and BHI had been used, since *E. coli* strains have been observed inducing virulence genes in BHI (Garsin et al., 2001); however, this was not part of the current analysis. An ORF is considered translated, when it is covered with at least one read per million mapped sequenced reads normalized to 1 kbp, ≥50% of the ORF is covered with RIBOseq reads, and the ribosomal coverage value (RCV) is at least 0.25 in both biological replicates. Promising candidates were verified by visual inspection using the Artemis genome browser (Rutherford et al., 2000).

### Bioinformatics Methods Used for *ano* Description

#### Prediction of σ^70^ Promoters

The region 300 bp upstream of the TSS of *ano* was searched for the presence of a σ^70^ promoter with the program BPROM (Softberry) (Solovyev and Tatarinova, 2011). The given LDF score is a measure of promoter strength, whereupon an LDF score of 0.2 indicates presence of a σ^70^ promoter with 80% accuracy and specificity.

#### Prediction of Shine-Dalgarno Sequences

Base-pairing to the anti-Shine-Dalgarno (SD) sequence is rated according to the ΔG° values given in Ma et al. (2002) in a sliding window of 9 up to 30 bp upstream of a start codon. The sum of all ΔG°-values for the optimal SD sequence in *E. coli* (uaaggaggu) is -9.6, but any sequence ≤-2.9 is considered a functional SD sequence.

#### Prediction of ρ-Independent Terminators

The regions 300 bp downstream of the stop codons of ECs2384 and *ano* were searched for the presence and folding energy of a ρ-independent terminator with the program FindTerm (Softberry) (Solovyev and Tatarinova, 2011).

#### Prediction of the Terminator Secondary Structure

The RNA sequence of the ρ-independent terminator predicted with FindTerm was submitted to the Mfold web server RNA Folding Form using default parameters to determine the secondary structure (Zuker, 2003).

#### Detection of Annotated Homologs

The AA sequence of the putative protein Ano was used to query the data base GenBank refseq with blastp using default parameters (Altschul et al., 1990).

#### PredictProtein

The AA sequence of Ano was submitted to the software PredictProtein (Yachdav et al., 2014). The results of PROFphd (prediction of secondary structures) (Rost and Sander, 1994), TMSEG (number of transmembrane helices) (Bernhofer et al., 2016), DISULFIND (number of disulfide bonds) (Ceroni et al., 2006), and LocTree2 (prediction of subcellular localization) (Goldberg et al., 2014) were examined in further detail.

#### Phylogenetic Tree Construction

The novel gene *ano* and the annotated genes ECs2384 and ECs2385 were phylostratigraphically analyzed to trace back the sequence evolution during species evolution. tblastn (NCBI, e-value cutoff ≤ 10^-10^, identity cutoff ≤ 50%) was used to search for homologous nucleotide sequences of EC2384 and ECs2385 in all genomic sequences of the nr database independent of their annotation status. Exemplary sequences within a broad range of sequence identities were downloaded. Multiple sequence alignments were conducted using MUSCLE implemented in MEGA6 (Tamura et al., 2013). The automated alignments were manually checked and adapted, where necessary. Homologs of *ano* and *ano*-sequences interrupted by stop codons were identified using a multiple alignment of the -3 frames of the respective homologs of ECs2385, which is the overlapping ‘mother’ frame for *ano*. The area, in which *ano* or *ano*-like fragments were located was translated to the AA sequence and aligned by multiple sequence alignment as before.

Phylogenetic trees of the strains and species examined were constructed according to Fellner et al. (2015). Briefly, a concatenated sequence of the housekeeping genes 16S rRNA*, atpD, adk, gyrB, purA*, and *recA* was used. The sequences were aligned using ClustalW in MEGA6. Columns with gaps or ambiguities were removed and the final dataset contains 8025 positions. The best nucleotide substitution model was searched using MEGA6. The final Maximum-Likelihood tree was calculated using Neighbor Joining and bootstrapped 1000-times. The best nucleotide substitution model for tree construction was identified to be the General Time Reversible model (GTR), assuming that a certain fraction of sites is evolutionarily invariable (+I, 20.34% sites). The non-uniformity of evolutionary rates among substitution sites was modeled using a discrete Gamma distribution with five rate categories (+G, parameter = 0.3102). The log likelihood value of the final tree was -61621.

## Results

### Detection of an Overlapping ORF Covered With RNAseq and RIBOseq Reads

**FIGURE 1:**
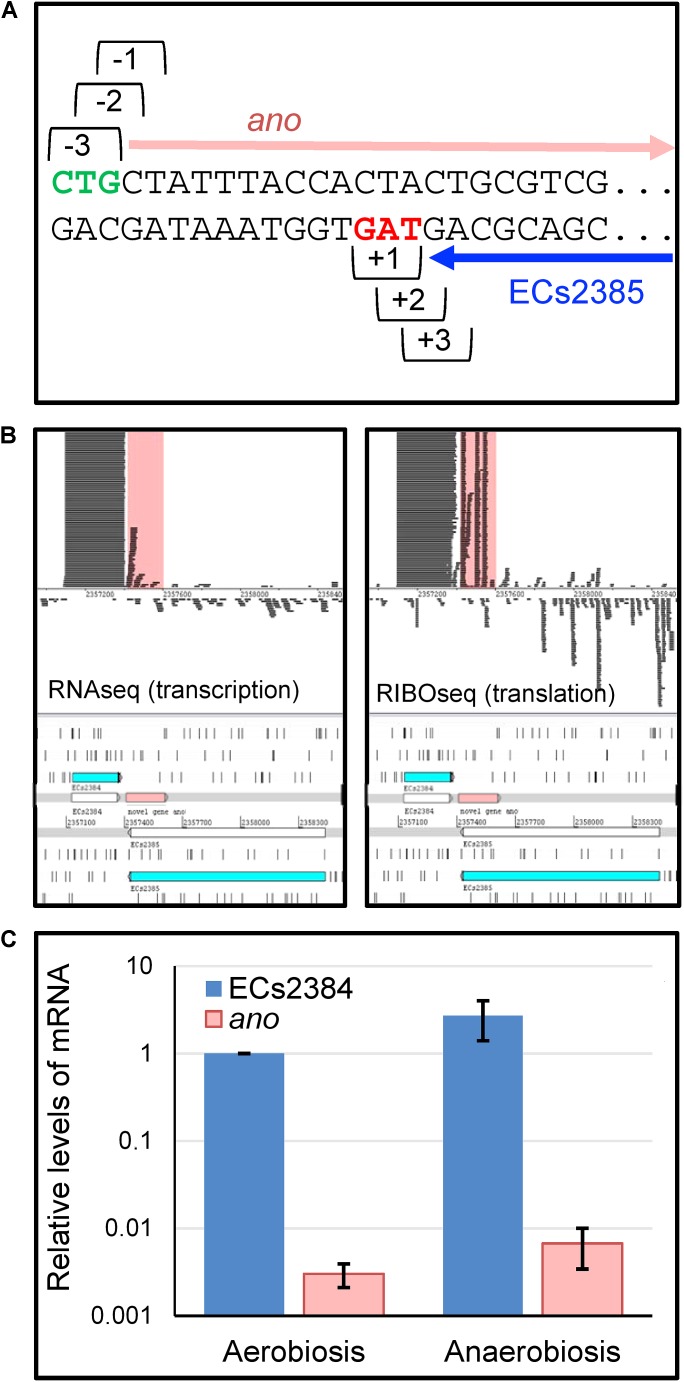
Reading frames, transcription, and translation of ECs2384, the overlapping gene *ano* and its mother gene ECs2385. **(A)** Definition of reading frames of the overlapping gene *ano* (frame –3) and its mother gene ECs2385 (frame +1). *ano* (pink arrow) overlaps the C-terminal part of ECs2385 (blue arrow). Green: putative start codon of *ano*, red: stop codon of ECs2385. **(B)** Visualization of RNAseq and RIBOseq reads of the *ano*/ECs2385 region with the upstream ECs2384. Using the genome browser Artemis, strand-specifically mapping reads of the growth condition LB at 37°C are shown (not to scale, since not all reads are shown). Both annotated genes, ECs2385 and ECs2384, are shown in blue, the overlapping gene *ano* is highlighted in pink as well as the reads mapping to *ano* in the upper panel. All three genes depicted show transcription and translation signals, although to a very different extent. **(C)** Relative mRNA-levels of ECs2384 and *ano*. Transcription levels were determined by qRT-PCR under the following conditions: 0.5×LB aerobically, OD_600_ of 0.5, and 0.5×LB anaerobically, OD_600_ of 0.5. The mRNA level of ECs2384 under aerobic conditions was set to 1 and used as reference. The experiment was performed in three biological replicates.

Strand-specific RNAseq and RIBOseq data sets at three different growth conditions (Hücker et al., 2017) were analyzed with regard to transcription and translation of ORFs antisense overlapping to annotated genes under aerobic growth conditions. Thereby, an ORF, later termed *ano*, overlapping in reading frame -3 (**Figure 1A**) to the annotated gene ECs2385 was discovered. The mother gene ECs2385 is annotated as a conserved hypothetical protein containing a transpeptidase domain, which covers 87% of this gene and has 99% blast similarity with a multi-strain L,D-transpeptidase of *E. coli* (NCBI, GenPept ID WP_096959586.1). ECs2385 is expressed under several conditions (R. Landstorfer, unpublished data). At all growth conditions, *ano* and ECs2385 are covered with RNAseq and RIBOseq reads, but to very different extents (**Figure 1B** and **Table 1**). In LB medium, *ano* shows the highest translation value. Furthermore, the translatability in LB is extremely high as indicated by a RCV of 18.2. In BHI medium at 37°C, transcription of *ano* is twofold increased, whereas translation is 3.5-fold reduced compared to LB. Even though the translatability is reduced 6.6-fold, the RCV is still clearly above the threshold of around 0.25 used to discriminate translated RNA from untranslated RNA (Neuhaus et al., 2017). At combined cold and osmotic stress, translation and translatability decrease massively (60- and 55-fold reduction compared to LB, respectively) (**Table 1A**). The annotated gene ECs2384 upstream of *ano*, which encodes a murein lipoprotein, is very highly transcribed and translated at all conditions investigated (**Figure 1B** and **Table 1B**). A qRT-PCR analysis confirms that ECs2384 is transcribed to a much higher extent than *ano*; the transcription of *ano* is about 330-fold lower under aerobic growth and about 400-fold lower under anaerobic growth, respectively (**Figure 1C**). The annotated mother gene ECs2385 is moderately expressed, showing the highest transcription at BHI stress and the highest translation value in LB (**Table 1C**).

**Table 1:** Transcription and translation of **(A)** *ano*, **(B)** ECs2384, and **(C)** ECs2385.

Condition	RPKM transcriptome^∗^	RPKM translatome^∗^	Ribosomal coverage value (RCV)^∗^	ORF coverage^∗^
**(A) *ano***
LB, 37°C	50	923	18.18	87%
BHI, 37°C	95	261	2.72	90%
BHI + 4% NaCl, 14°C	46	16	0.33	58%
**(B) ECs2384**
LB, 37°C	39792	68550	1.83	100%
BHI, 37°C	16006	16979	1.06	100%
BHI + 4% NaCl, 14°C	10197	2892	0.32	100%
**(C) ECs2385**
LB, 37°C	16	21	1.42	58%
BHI, 37°C	13	8	0.73	56%
BHI + 4% NaCl, 14°C	49	7	0.15	61%

*The RPKM values of the transcriptome and translatome data for the novel gene, the overlapping annotated gene and the upstream-annotated gene are listed. The ribosomal coverage value, a measure of the translatability, was calculated by the ratio of RPKM translatome to RPKM transcriptome. ORF coverage gives the percentage of gene sequence, which is covered by RIBOseq reads. ^∗^Mean values of the two biological replicates are shown*.

### Determination of the Transcriptional Start Site and Promoter Activity

**FIGURE 2:**
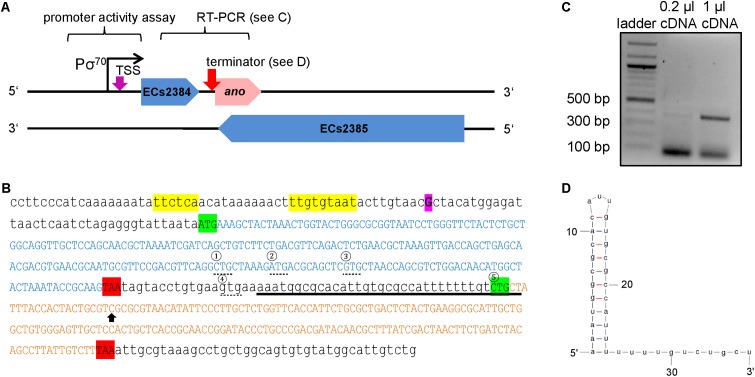
Genomic organization of ECs2384 and *ano* overlapping to its mother gene ECs2385. **(A)** The annotated genes ECs2384 and ECs2385 are depicted in blue and the novel OLG *ano* is depicted in pink. The genes and the distances are drawn to scale. The predicted σ^70^ promoter, the experimentally determined transcription start site (TSS, purple arrow), and the predicted ρ-independent terminator between ECs2384 and *ano* (pink arrow) are sketched. DNA stretches, which were used for promoter activity assay and RT-PCR, are indicated. **(B)** DNA sequence of ECs2384 and *ano*. The sequence of the annotated gene ECs2384 is written in blue capital letters. The sequence of *ano* is written in pink capital letters. The start codons are highlighted in green and the stop codons in red. The four alternative upstream start codons of *ano* are marked by a dashed line and numbered consecutively. The fifth start codon, we propose as the correct one (see text). The TSS detected by 5′ RACE is highlighted in purple. The predicted σ^70^ promoter is colored in yellow and the predicted ρ-independent terminator is underlined. Black arrow: the nucleotide C was changed to an A in order to create a stop codon (compare to **Figure 4**). **(C)** Agarose gel picture of the RT-PCR product ECs2384-*ano*. A 100 bp DNA ladder (NEB) was used as a size standard. A primer pair was used for PCR with EHEC cDNA as template spanning the sequences of ECs2384 and *ano*. The gel shows a product of the anticipated size of 380 bp, indicating that ECs2384 and *ano* are transcribed as a bicistronic mRNA. **(D)** Secondary structure of the ρ-independent terminator predicted using Mfold.

**FIGURE 3:**
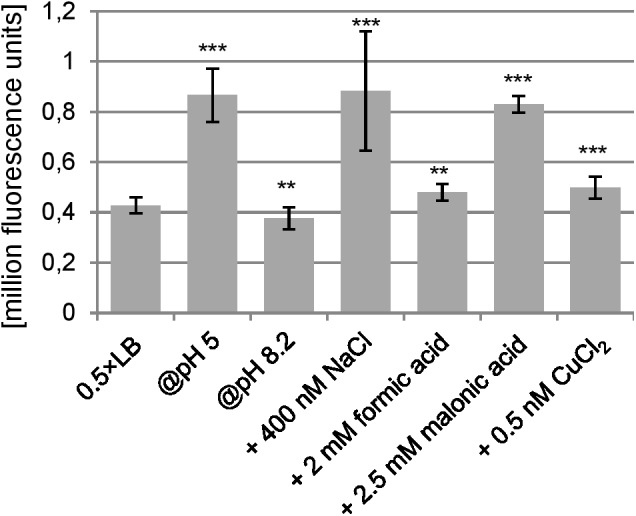
Promoter activity of the region upstream of the TSS under anaerobic conditions. At an OD_600_ of 0.5, the fluorescence caused due to the pProbe-NT-PromoterTSS plasmid was measured. As negative control, the fluorescence of *Escherichia coli* transformed with pProbe-NT was also determined, which was 289 ± 110 (not shown in the diagram). The fluorescence value of pProbe-NT-PromoterTSS was at all conditions significantly higher than the vector control (*p* < 0.001). Significant changes between 0.5×LB and investigated stress conditions were calculated by Student’s *t*-test and marked with asterisks (^∗∗^*p* < 0.01, ^∗∗∗^*p* < 0.001).

Transcriptional start site determination using 5′ RACE resulted in a single signal 324 bp upstream of the proposed *ano* start codon (**Figure 2A**). This TSS is also 38 bp upstream of the annotated gene ECs2384 and fits well to a σ^70^ promoter 9 bp upstream of the TSS predicted by the software BPROM (**Figure 2B**). An LDF-score of 5.56 indicates high promoter strength, which was confirmed by assaying the region upstream of the TSS using an EGFP-reporter plasmid, as a high fluorescence intensity was measured at all anaerobic growth conditions investigated (**Figure 3**). Compared to the vector control, the fluorescence intensity is about 1000-fold increased. In addition, differential promoter activity between the tested stress conditions occurs: the promoter activity in pH 5 and in LB medium supplemented with 400 mM NaCl or 2.5 mM malonic acid is twofold increased, compared to plain LB. A high promoter activity was found at aerobic growth as well (data not shown).

Reverse transcription polymerase chain reaction (RT-PCR) using a primer pair spanning both genes, ECs2384 and *ano*, resulted in a PCR product of 380 bp, which indicates that the two genes are co-transcribed as a bicistronic mRNA (**Figure 2C**). The upstream sequence of *ano* contains a SD-like sequence, aatggcgca, with a ΔG° of -2.2 and in range of naturally occurring SD sequences (i.e., 21 bases upstream the proposed start codon 5). Both regions downstream of the ECs2384 and *ano* stop codons were investigated for the presence of a ρ-independent terminator using the software FindTerm. The intergenic sequence between ECs2384 and *ano* contains a terminator with a folding energy of -20.8 (**Figures 2B,D**). This terminator explains why ECs2384 is transcribed to a much higher extent than *ano* (**Table 1**), even though the two genes are organized in an operon. Most transcription events probably stop at this terminator and only a monocistronic ECs2384 mRNA is produced. However, termination is not 100% efficient and, thus, some transcription events produce a bicistronic mRNA comprising both ECs2384 and *ano*. Interestingly, the upregulation of the upstream ECs2348 is in the same range as upregulation of *ano* (between 2- and 3-fold, **Figure 1C**). This suggests that a given fraction of RNA is read through the terminator, producing the bicistronic mRNA. In contrast, downstream of *ano*, no ρ-independent terminator is predicted. Since it is impossible to predict ρ-dependent terminators computationally (Peters et al., 2011), it remains unclear how termination of transcription downstream of *ano* is regulated.

### Properties of the Hypothetical Protein Ano

Five potential start codons are present in the ORF under discussion (**Figure 2B**, circles 1 to 5): the first start codon CTG would lead to a 101 AA protein, the second start codon ATG to a 98 AA protein, the third start codon GTG to a 94 AA protein, a fourth start codon GTG to a 74 AA protein and the fifth start codon CTG would produce a 62 AA protein. The *ano* sequence was cloned upstream of EGFP into the plasmid pEGFP. Plasmids for the possible start codons 1, 2, 3, and 5 were constructed and transformed into *E. coli* Top10. After induction with 10 mM IPTG, Ano-EGFP fusion proteins were expected to be expressed, indicated by an increase in fluorescence intensity. Plasmids with the translationally arrested *ano^∗^* ORF (black arrow in **Figure 2B**, see also below) were used as negative controls. As expected, the empty pEGFP plasmid (positive control) leads to a high increase of fluorescence intensity after induction (data not shown). The fluorescence of constructs using the putative start codons 1, 2, and 3 was not distinguishable from the background fluorescence of *E. coli*, either uninduced or induced (**Table 2**). Only *ano* start codon 5 caused a 3.7-fold increase of fluorescence intensity compared to the uninduced culture and had the highest fluorescence value for all *ano* start codons tested, indicating translation of the fusion protein. In contrast, induction of *ano^∗^* using start codon 5 did not change the observed low fluorescence intensity.

**Table 2:** Expression of a C-terminally Ano-EGFP fusion protein.

Sample	Fluorescence 0 mM IPTG	Fluorescence 10 mM IPTG	Significance
*E. coli* Top10 + pEGFP-*ano*-Start1	0	0	N/A
*E. coli* Top10 + pEGFP-*ano^∗^-*Start1	0	1124 ± 152	N/A
*E. coli* Top10 + pEGFP-*ano*-Start2	0	0	N/A
*E. coli* Top10 + pEGFP-*ano^∗^-*Start2	0	0	N/A
*E. coli* Top10 + pEGFP-*ano*-Start3	0	0	N/A
*E. coli* Top10 + pEGFP-*ano^∗^-*Start3	0	291 ± 56	N/A
*E. coli* Top10 + pEGFP-*ano*-Start5	883 ± 703	3305 ± 236	^∗∗∗^
*E. coli* Top10 + pEGFP-*ano^∗^-*Start5	466 ± 312	397 ± 169	–
*E. coli* Top10 + pEGFP-ECs2385	527 ± 430	8662 ± 2444	^∗∗∗^

*Escherichia coli Top10 was transformed with the different pEGFP-*ano* plasmids and expression of the fusion protein was induced using 10 mM IPTG. OD_*600*_ was adjusted to 0.6 and fluorescence was measured. Fluorescence values of empty *E. coli* Top10 were subtracted (causing zero values for some readings). The experiment was performed in triplicate. The first three potential start codons had zero fluorescence, when not induced. Therefore, calculation of significance was meaningless (N/A) for those. Significant changes between induced and uninduced cultures were calculated by two-tailed Student’s *t*-test (^∗∗∗^*p* < 0.001)*.

Since ECs2385 is annotated as a hypothetical protein only, expression of an ECs2385-EGFP fusion protein was tested additionally and its induction leads to an increase of fluorescence intensity as well (**Table 2**).

The derived AA sequence of Ano was analyzed using PredictProtein. The secondary structure consists mainly of loops and hydrophilic α-helices, but no membrane helices were predicted. One disulfide bond was predicted, and the protein might be secreted. A blastp search for annotated homologs in other bacteria did not obtain any hit.

### Phenotype of *ano* Under Anaerobic Conditions

**FIGURE 4:**
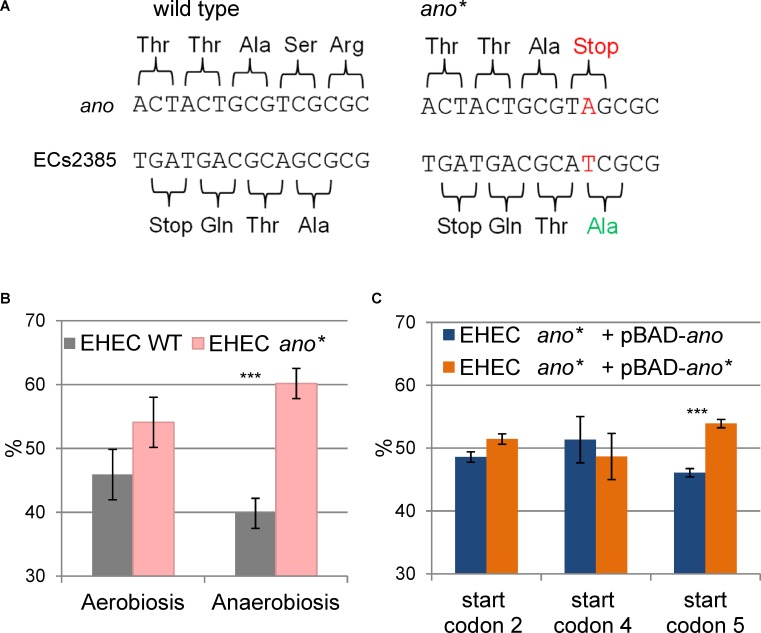
Construction and phenotype of the translationally arrested mutant *ano^∗^*. **(A)** Introduction of a point mutation (TCG to TAG) resulted in a premature stop codon in the N-terminal sequence of *ano*. The point mutation is located downstream of the ECs2384 stop codon and does not change the amino acid sequence of the antisense overlapping mother gene ECs2385. In addition, a cut site for the restriction enzyme *Hga*I happened to be deleted by this mutation. **(B)** Phenotype of *ano^∗^*. The abundance in percent of EHEC wild type (WT) and EHEC *ano^∗^* are shown after competitive growth in 0.5×LB medium aerobically and anaerobically. Wild type and mutant were mixed in equal numbers and their ratio was determined by Sanger sequencing after 18 h of competitive growth. The mutant has a significant growth advantage under anaerobiosis. The experiment was performed in biological triplicates. Significant changes were calculated by Student’s *t*-test (^∗∗∗^*p* < 0.001). **(C)** Complementation of EHEC *ano^∗^ in trans.* The abundance in percent of EHEC *ano^∗^* with a plasmid either having or not having a functional copy of *ano* shown after competitive growth anaerobically in 0.5×LB medium. EHEC *ano^∗^* was transformed with the plasmid pBAD carrying the intact *ano* ORF (using either start codons 2, 4, or 5) and competitive growth was performed against EHEC *ano^∗^* + pBAD-*ano^∗^*. The plasmid was induced using 0.002% arabinose. Only the use of start codon 5 partly restores the phenotype of the wild type. The experiment was performed in biological triplicates. Significant changes were calculated by Student’s *t*-test (^∗∗∗^*p* < 0.001).

In order to search for a phenotype of *ano*, a strand-specific translationally arrested mutant EHEC *ano^∗^* was created by changing a single nucleotide of the eighth *ano* codon (counting from putative start codon 5, **Figure 2B**) leading to a premature stop codon. The point mutation introduced does not change the AA sequence of the overlapping gene ECs2385, because the mutation is synonymous for the +1 reading frame (**Figure 4A**).

Competitive growth experiments were performed with equal inoculation ratios of EHEC wild type and EHEC *ano^∗^* to search for a phenotype. When the cultures were incubated aerobically, the ratio between wild type and mutant did not change significantly (**Figure 4B**). In contrast, anaerobic incubation resulted in a small but significant and consistent growth advantage of EHEC *ano^∗^*. The anaerobic competitive growth experiment was also performed at several other stress conditions and the observed phenotype was similar to plain LB, i.e., the mutant strain showed a small growth advantage compared to the wild type (**Supplementary Figure S1**). Interestingly, transcription of both *ano* and of its upstream gene ECs2384 are increased at anaerobiosis compared to aerobic incubation 2.2- and 2.7-fold, respectively (**Figure 1C**).

Although the translational arrest of *ano* leads to a weak phenotype only, a complementation *in trans* was performed, transforming EHEC *ano^∗^* with the plasmid pBAD-*myc/His-*C carrying an intact *ano* ORF under the control of an arabinose inducible promoter. As a negative control, the same mutant was also transformed with a plasmid containing the translationally arrested *ano^∗^* ORF. Competitive growth experiments were performed anaerobically as described before. Complementation plasmids for three different putative *ano* start codons (**Figure 2B**) were tested. Plasmids using the putative start codons 2 (ATG) and 4 (GTG) did not show significant changes of the ratio of *ano* over *ano^∗^* (**Figure 4C**). In contrast, when putative start codon 5 (CTG) was used, induction resulted in a small but consistent growth disadvantage of the complemented strain after competitive growth compared to translationally arrested *ano^∗^.* However, since the observed difference between wild type and mutant is larger (**Figure 4B**), only a partial complementation was possible.

### Phylogeny of ECs2384 and the OLG Pair *ano*/ECs2385

The phylogenetic distribution of the annotated genes ECs2384, ECs2385, and of the novel gene *ano* was investigated to estimate the relative age of these genes. Homologous sequences were searched using tblastn applying an e-value cutoff of ≤ 10^-10^ and their annotation status was verified with blastp. Annotated homologs of the ‘mother’ gene ECs2385 are present in many bacterial species (**Supplementary Figure S2**).

The upstream gene ECs2384 is predominantly annotated in species of the order *Enterobacteriales*. Using an e-value of ≤ 10^-10^, which is a stringent cut-off, tblastn identifies only one homolog of ECs2384 outside *Enterobacteriales*, in *Aeromonas*. However, blastp shows that the gene is annotated in additional 11 genera. Seven of them are γ-proteobacteria of the order *Vibronales*, *Pseudomonales*, *Aeromonales* and an unclassified *Gallaecimonas*. Remaining hits belong to the genera *Achromobacter*, *Mycobacterium*, *Beauveria*, and *Enterococcus*. If present, the AA sequence of ECs2384 is highly conserved, showing only very few AA substitutions (**Supplementary Figure S3**).

**FIGURE 5:**
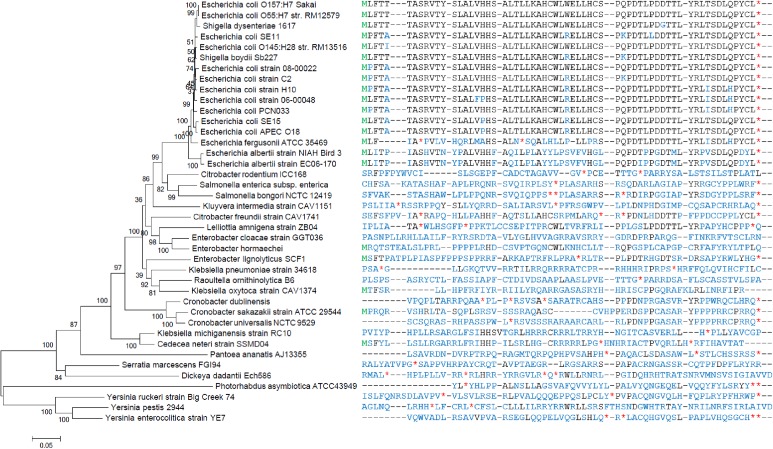
Phylogenetic tree and sequence alignment of Ano. The phylogenetic tree on the left was constructed for representative species possessing homologs of *ano* or ECs2385, antisense overlapping to each other. The tree is based on a concatemer of 16S rRNA, *atpD*, *adk*, *gyrB*, *purA*, and *recA.* On the right, the different amino acid sequences of Ano (if present) are aligned (black). Translated start codons are colored in green and untranslated stop codons in red (^∗^). Variable regions with no detectable amino acid homology to Ano are colored in blue.

Ano homologs have not been annotated in any bacterium. Intact homologs (i.e., without internal stop codons) of Ano are only found in *E. coli* and *Shigella* strains (**Figure 5**) with only one exception: *E. coli* FHI92 (LM997172.1) contains an *ano* sequence with an internal stop codon. Homologs (or fragments thereof) with lower similarity were found in *E. albertii* and *E. fergusonii*. The sequence in both strains of *E. albertii* is intact and extended only at the 3′ end. The sequence in *E. fergusonii* has an internal variable region containing stop codons and, thus, is probably dysfunctional.

In summary, the annotated genes ECs2384 and ECs2385 are conserved in *Enterobacteriaceae*, whereas *ano* only has homologs in the *Escherichia/Shigella* clade, indicating a younger evolutionary age.

## Discussion

### Is *ano* a Protein-Coding Gene?

In bacteria, regulatory RNAs are frequently encoded antisense to annotated protein-coding genes (Dornenburg et al., 2010; Georg and Hess, 2011) whereas only very few examples of non-trivial protein-coding overlapping genes are known (Tunca et al., 2009; Fellner et al., 2014, 2015). Thus, instead of being an mRNA, *ano* might encode a novel ncRNA. Coverage of the ORF with RNAseq reads (**Figure 1B** and **Table 1A**) and the presence of a promoter (**Figure 3**) would support both, an ncRNA and a protein-encoding gene. However, several observations contradict the hypothesis that *ano* is solely an ncRNA. First, the ORF is clearly covered by RIBOseq reads indicating active translation. RIBOseq has been used successfully in the past to detect translation of non-annotated genes in eukaryotes (Fritsch et al., 2012; Bazzini et al., 2014; Van Damme et al., 2014) and prokaryotes (Neuhaus et al., 2016; Baek et al., 2017). It is highly unlikely that such a high RIBOseq signal is caused by contaminating RNA binding proteins (adsorbing RNA molecules and, thus, causing a carry-over in the RIBOseq preparation) or *ano* RNA randomly bound to ribosomes (Liu and Qian, 2016). Furthermore, start and stop regions of the RIBOseq signal fit very well to the *ano* ORF (**Figure 1B**). Second, the translatability of *ano* RNA in LB medium is exceptionally high (**Table 1A**), since we found an RCV of ca. 18. Short annotated EHEC genes only have a mean RCV of 1.55 at the same conditions (Hücker et al., 2017). Neuhaus et al. (2017) report that the mean RCV of tRNAs, which are also not translated similar as ncRNAs, is 0.06 and they suggest that an ORF with an RCV of at least 0.3 can be considered a protein-coding gene in their data. Third, to our knowledge, ncRNAs are not transcribed as a bicistronic RNA together with a protein-coding gene in prokaryotes (**Figure 2C**). Fourth, expression of an Ano-EGFP fusion protein was possible (**Table 2**) which corroborates the RIBOseq data, i.e., the *ano* RNA can be translated into an amino acid sequence. Fifth, the change of a single nucleotide in the *ano* sequence producing a translationally arrested mutant had a clear phenotype. It is considered very unlikely that a single nucleotide change in an ncRNA will lead to a phenotype. Antisense ncRNAs typically regulate the expression of target mRNAs by base pairing through limited and imperfect complementarity over a stretch of several nucleotides such that a single base substitution will hardly abolish this pairing (Bobrovskyy and Vanderpool, 2013). It is suggested that this fivefold evidence renders the hypothesis that *ano* is a protein-coding gene a convincing interpretation of the data.

However, there are potential objections to the conclusion that *ano* is an overlapping gene. First, a strong SD sequence is missing and, second, ECs2385 could be wrongly annotated. Concerning the SD sequence, only a weak SD-like sequence with ΔG° of -2.2 could be found. A ΔG° below -2.9 is considered indicating a functional SD sequence (Ma et al., 2002). However, according to our analysis, *ano* is a recently evolved gene (see below) and a weak SD sequence would fit well to this notion. Even the absence of a SD sequence is not excluding protein production, since *E. coli* has about 39% of non-SD-led genes (Chang et al., 2006). Similarly, the SD score has little predictive power for *E. coli* in locating protein-coding ORFs (Friedman et al., 2017). Even more surprising, it was shown that the anti-Shine-Dalgarno region of the 16S rRNA is dispensable for the correct selection of translational start (Melancon et al., 1990). Taken together, an absent or weak SD sequence certainly does not preclude protein-expression in *E. coli*. However, the mother gene ECs2385 is annotated as hypothetical in EHEC and if ECs2385 is not a protein-coding gene, *ano* could not be considered as overlapping gene. In former times, there have been some doubts that hypothetical (and especially short) genes are “real” genes (Skovgaard et al., 2001; Ochman, 2002). Nowadays, most researchers believe that hypothetical genes annotated by current genome annotation programs are indeed protein coding (Storz et al., 2014; Baek et al., 2017). There is strong evidence that ECs2385 is a protein-coding gene. First, the derived amino acid sequence of this gene is highly conserved over the family Enterobacteriaceae. There is no ECs2385 sequence known in this family that is interrupted by a stop codon. Therefore, the protein encoded is under selection and must confer some fitness increase. Second, ECs2385 has an annotated transpeptidase motif, which indicates enzymatic functionality. Third, ECs2385 can be translated to a stable amino acid sequence (**Table 2**). Taken together, the objection that ECs2385 is not a protein-coding gene appears to be quite weak.

### Does *ano* Use the Rare Start Codon CTG?

Surprisingly, the experimental data presented supports the fifth in-frame start codon of *ano*, which is a rare CTG instead of the more common ATG or GTC start codons. According to the genetic code table 11^1^, ATG is the most frequent start codon in bacteria. In addition, GTG and TTG can be used as well and only rare cases of CTG and ATH (H = T, C, A) start codons have been reported. Five putative in-frame start codons are present (in order CTG, ATG, GTG, GTG, and CTG; **Figure 2B**) leading to potential *ano* variants, all of which have been translationally arrested by introduction of a stop codon (**Figures 2B**, **4A**). Induction of expression of an Ano-EGFP fusion protein was only possible when using the fifth start codon, which is a CTG (**Table 2**). Furthermore, partial complementation of the *ano^∗^* phenotype was achieved using this downstream CTG start codon, but was not possible using any other start codon (**Figure 4C**). These results indicate that the second CTG (overall fifth) and not the canonical ATG (overall second) probably is the correct start codon of *ano*. In *E. coli*, only one other gene, the plasmid borne *repA*, is confirmed to start with CTG (Spiers and Bergquist, 1992). Interestingly, RIBOseq data of *E. coli* using the antibiotic tetracycline to stall translation at the translational start site (i.e., the start codon) indicates three additional genes with CTG start codons as alternative start sites (Nakahigashi et al., 2016). Also in our data, the bulk of RIBOseq reads for the *ano* ORF start at the CTG start codon (**Figure 1** highlights *ano* starting at this particular CTG). Finally, the S12 ribosomal protein *rpsL* of *Deinococcus deserti* starts with CTG (Baudet et al., 2010). New methods, like sequencing of translation initiation (QTIseq) (Gao et al., 2015) and N-terminal proteomics (Van Damme et al., 2014; Willems et al., 2017), may additionally confirm initiation by rare start codons. However, only one point mutation is required to change the rare CTG start codon of *ano* into a start codon used more frequently.

### Origin of the Novel Overlapping Gene *ano*

Phylostratigraphic analyses indicate that the mother ORF ECs2385 antisense to the overlapping gene *ano*, as well as the upstream gene ECs2384 are conserved genes that originated before the *Enterobacteriales* diversified (**Supplementary Figures S2**, **S3**). The upstream gene ECs2384 is mainly restricted to the *Enterobacteriales* and some further related γ-proteobacteria. However, the mother gene ECs2385 is widely distributed among many bacterial families. The distant relatives do not harbor an *ano* homolog. In contrast, *ano* is taxonomically strongly restricted and probably evolved after the separation of the *Escherichia/Shigella* clade from other *Enterobacteriaceae* (**Figure 5**). *Ano* overlaps antisense to ECs2385, certainly causing constraints in the evolution of both genes (Johnson and Chisholm, 2004; Lillo and Krakauer, 2007). However, such a constraint is not apparent for the ECs2385 sequence. Both parts, overlapping and non-overlapping to *ano*, are highly conserved with only few amino acid substitutions for ECs2385 homologs in the *Escherichia/Shigella* clade. Interestingly, *ano* is encoded in frame -3 relative to ECs2385 and this combination provides the highest freedom for variation in two evolutionary coupled overlapping genes (Mir and Schober, 2014).

The formation of *ano* via overprinting in the first place would not require a novel promoter. The annotated gene ECs2384 is transcribed to a high extent anyway (**Table 1B**) and transcription is usually terminated at a downstream ρ-independent terminator (**Figures 2A,B**). Now, the RNA polymerase may occasionally read through a ρ-independent terminator and a longer mRNA will be produced (**Figure 2C**). This extended mRNA carries the small ORF *ano*, which may have originated by several point mutations. Next, this ORF now must be translatable into a stable protein. Such young genes certainly are volatile and will get lost if they do not confer a fitness increase in an environment experienced by the bacterium at least once in a while (Huvet and Stumpf, 2014). However, why should an antisense nucleotide sequence determined by the nucleotide sequence of an overlapping protein-coding gene form a functional protein? Some features of an overlapping ORF must pre-exist which enable the *ab initio* formation of a functional protein in the first place, but such features need to be explored by further research.

In case the novel protein will lead to some fitness advantage, at least under certain conditions, the novel ORF will eventually become fixed and evolve further under positive selection (Carvunis et al., 2012). In *E. coli* O157:H7 Sakai, *ano* exhibits a small, significant phenotype only under anaerobiosis (**Figure 4B**), clearly demonstrating a cellular impact of Ano under certain conditions. However, that phenotype confers a negative fitness value under anaerobic condition and with the same trend in aerobic condition, not a positive one. While direct or indirect positive functions of *ano* are expected to exist elsewhere since this overprinted gene was fixed in the *E. coli/Shigella* clade, it is impossible at this point to speculate on such potential functions based on the data available. Maybe, since Ano is a short protein with low abundance, it is adjusting properties of existing cellular systems like membranes (Hemm et al., 2008) or metabolic pathways. Clearly, a functional characterization of Ano requires additional studies.

## Author Contributions

SMH, SS, and KN designed and planned the study and wrote the manuscript. SMH performed the 5′ RACE experiments, promoter activity assays, competitive growth and complementation experiments, cloned the mutant *ano^∗^*, and performed the qRT-PCR. IA-S performed the expression of the Ano-EGFP fusion proteins. SV did the phylostratigraphic analysis of the overlapping gene pair *ano/*ECs2385 and the annotated gene ECs2384.

## Conflict of Interest Statement

The authors declare that the research was conducted in the absence of any commercial or financial relationships that could be construed as a potential conflict of interest.

## Footnotes

1 https://www.ncbi.nlm.nih.gov/Taxonomy/Utils/wprintgc.cgi#SG11

## Supplementary Material

FIGURE S1 Competitive growth of EHEC wild type against EHEC *ano^∗^* at anaerobic conditions in the presence of several stressors. Wild type and mutant were mixed in equal numbers and after 18 h incubation at different growth conditions their abundance was determined by Sanger sequencing. Significant changes between complementation and mutant were calculated by Student’s *t*-test (^∗∗^*p* < 0.05, ^∗∗∗^*p* < 0.001).

FIGURE S2 Phylogenetic tree of species with ECs2385 homologs. Possible start codons are colored in green and stop codons in red (^∗^). Variable regions are colored in blue. The pink arrow indicates the position of *ano*.

FIGURE S3 Phylogenetic tree of species with ECs2384 homologs. Possible start codons are colored in green and stop codons in red (^∗^). Variable regions are colored in blue.

TABLE S1 Bacterial strains and plasmids used in this study.

TABLE S2 Oligonucleotides used in this study. Restriction enzyme cut sites are highlighted in bold.
