# Supplementary figures and images for: The Novel Anaerobiosis-Responsive Overlapping Gene ano Is Overlapping Antisense to the Annotated Gene ECs2385 of Escherichia coli O157:H7 Sakai
Source: Front Microbiol. 2018 May 14;9:931. doi: 10.3389/fmicb.2018.00931 (PMC5960689; doi:10.3389/fmicb.2018.00931)

Figure S1

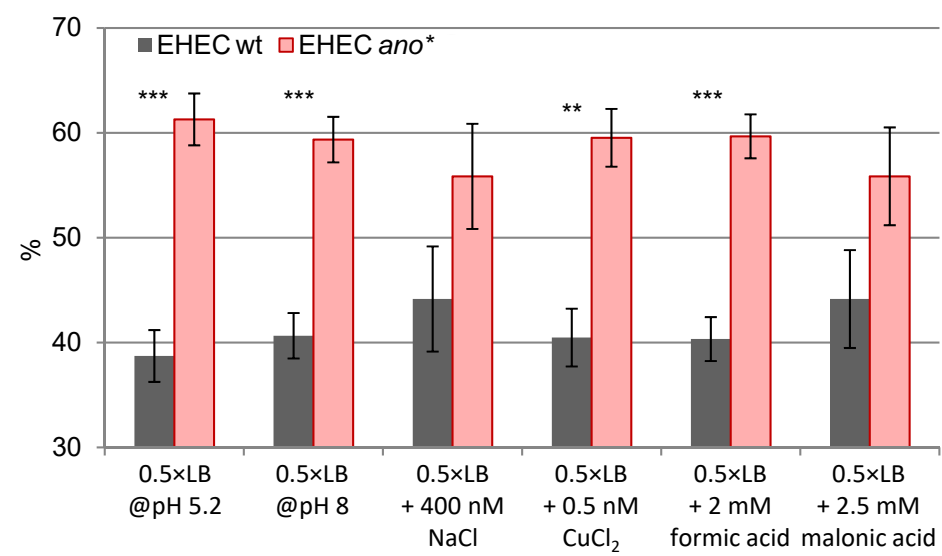

Supplement: FIGURE S1 — Competitive growth of EHEC wild type against EHEC ano∗ at anaerobic conditions in the presence of several stressors. Wild type and mutant were mixed in equal numbers and after 18 h incubation at different growth conditions their abundance was determined by Sanger sequencing. Significant changes between complementation and mutant were calculated by Student’s t-test (∗∗p < 0.05, ∗∗∗p < 0.001). [file Image_1.PDF]

Figure S2

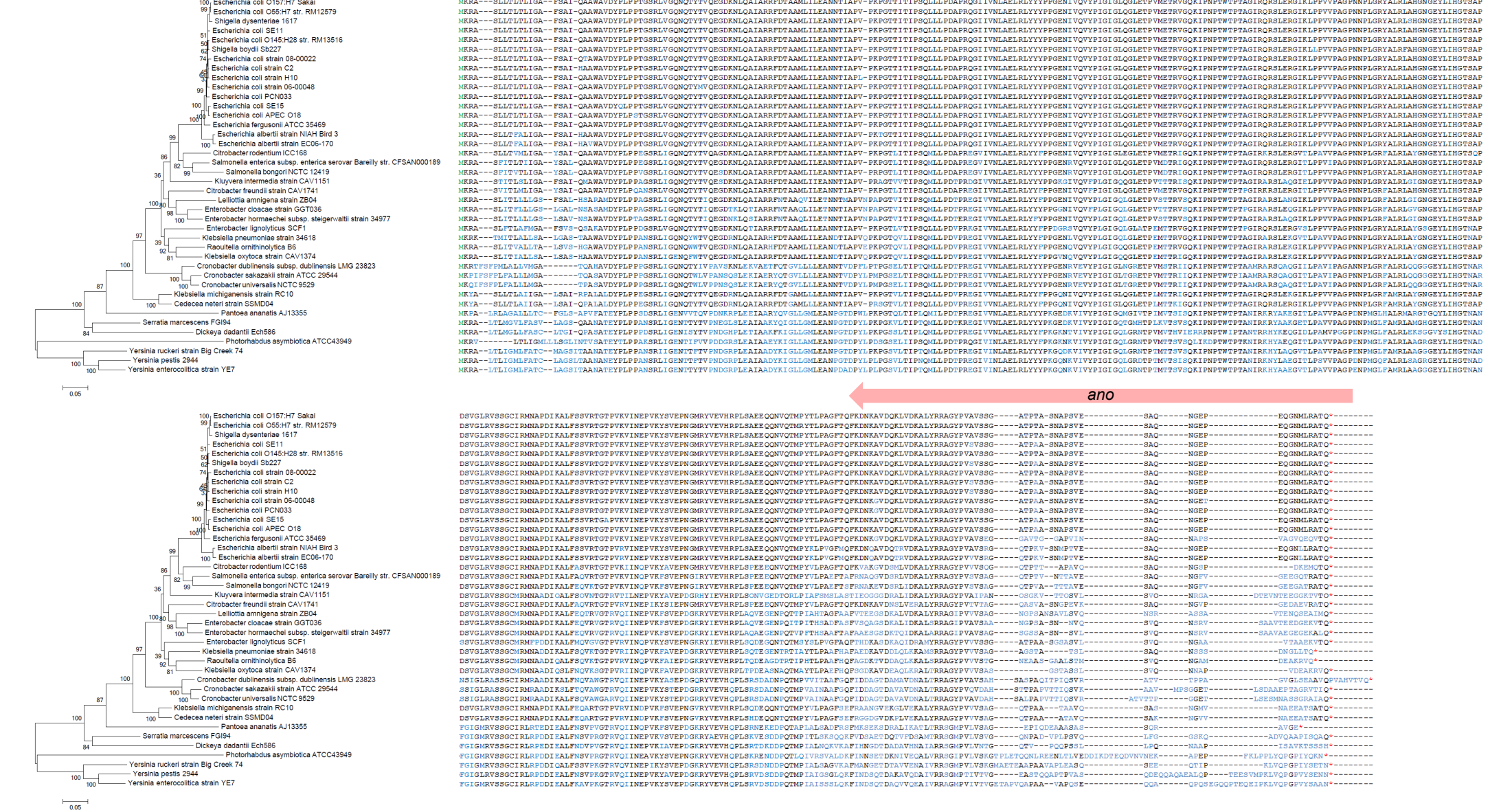

Supplement: FIGURE S2 — Phylogenetic tree of species with ECs2385 homologs. Possible start codons are colored in green and stop codons in red (∗). Variable regions are colored in blue. The pink arrow indicates the position of ano. [file Image_2.PDF]

### Figure S3

# ECs2384

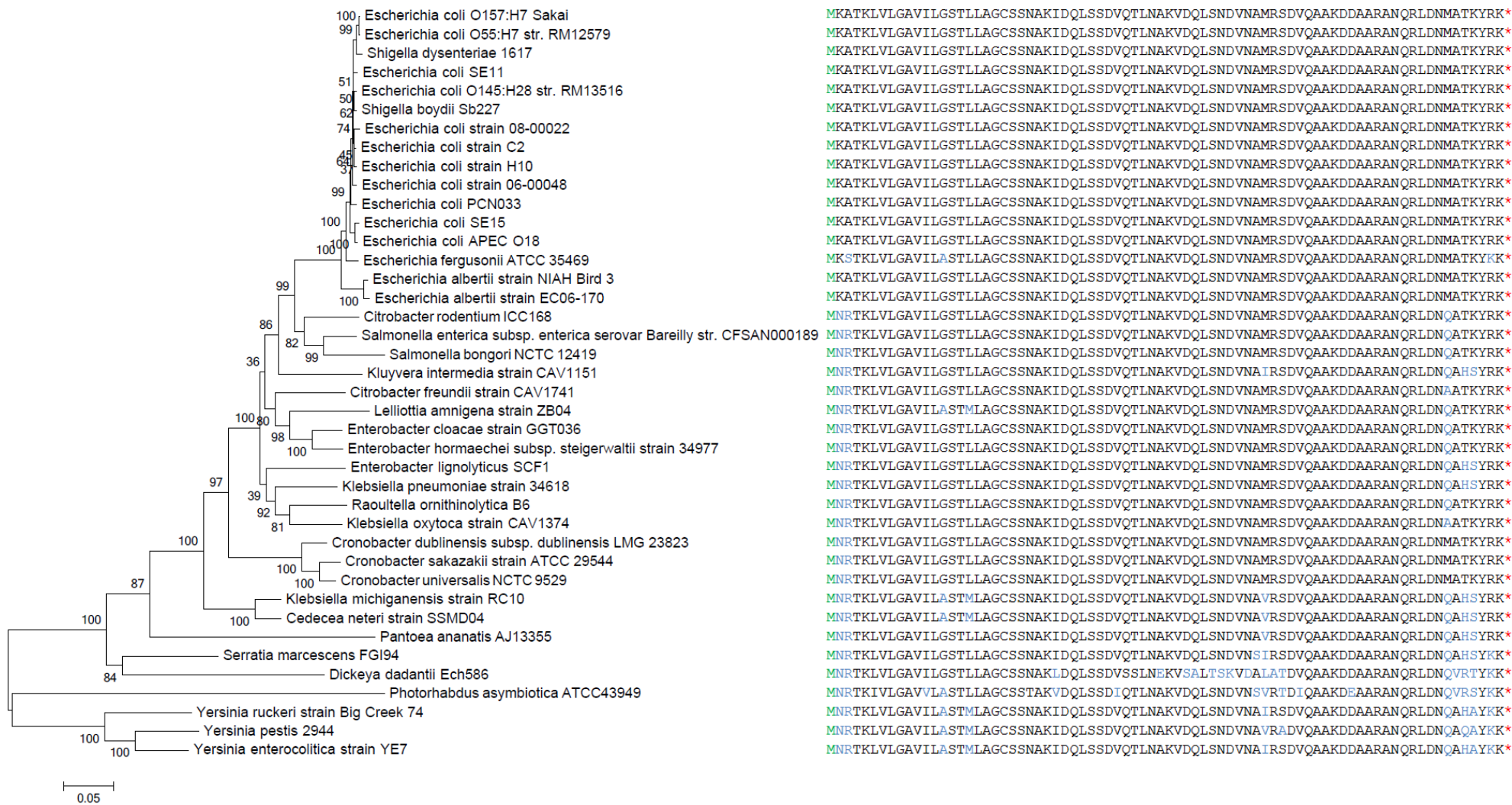

Supplement: FIGURE S3 — Phylogenetic tree of species with ECs2384 homologs. Possible start codons are colored in green and stop codons in red (∗). Variable regions are colored in blue. [file Image_3.PDF]
