# Supplementary material for: The Novel Anaerobiosis-Responsive Overlapping Gene ano Is Overlapping Antisense to the Annotated Gene ECs2385 of Escherichia coli O157:H7 Sakai
Source: Front Microbiol. 2018 May 14;9:931. doi: 10.3389/fmicb.2018.00931 (PMC5960689; doi:10.3389/fmicb.2018.00931)
Supplement: TABLE S1 — Bacterial strains and plasmids used in this study. [file Table_1.DOCX]

| strain or plasmid | characteristics | reference |
| --- | --- | --- |
| *Escherichia coli* O157:H7 Sakai (EHEC) | wild type, outbreak strain | Hayashi et al., 2001 |
| *Escherichia coli* O157:H7 Sakai ∆*ano* | translational arrested mutant of *ano* | this study |
| *Escherichia coli* Top10 | F-, *mcrA*, Δ(*mrr-hsdRMS-mcrBC*), φ80*lacZ*ΔM15, Δ*lacX*74, *nupG*, *recA*1, *araD*139, Δ(*ara-leu*)7697, *galE*15, *galK*16, rpsL(Str^R^), *endA*1, λ^-^ | Invitrogen |
| pProbe-NT | pBBR1 replicon, *gfp* reporter, Km^R^ | Miller et al., 2000 |
| pProbe-NT-PromotorTSS | 300 bp upstream of *ano* TSS | this study |
| pEGFP | pUC ori, P_lac_, *egfp* reporter, Amp^R^ | CLONTECH Laboratories |
| pEGFP-*ano*_start1 | C-terminal EGFP-fusion protein | this study |
| pEGFP-*ano**_start1 | C-terminal EGFP-fusion protein | this study |
| pEGFP-*ano*_start2 | C-terminal EGFP-fusion protein | this study |
| pEGFP-*ano**_start2 | C-terminal EGFP-fusion protein | this study |
| pEGFP-*ano*_start3 | C-terminal EGFP-fusion protein | this study |
| pEGFP-*ano**_start3 | C-terminal EGFP-fusion protein | this study |
| pEGFP-*ano*_start5 | C-terminal EGFP-fusion protein | this study |
| pEGFP-*ano**_start5 | C-terminal EGFP-fusion protein | this study |
| pEGFP-ECs2385 | C-terminal EGFP-fusion protein | this study |
| pSLTS | derivate of pKDTS | Kim et al., 2014 |
| pTS2Cb | derivate of pUC19, I-SceI, Amp^R^, Cm^R^ | Kim et al., 2014 |
| pTS2Cb-*ano** | premature stop codon in *ano* | this study |
| pBAD/*Myc*-*His*-C | derivate of pBR322, *araBAD* promoter, *myc C*-tag and his-tag fusion, Amp^R^ | Invitrogen |
| pBAD-*ano*_start2 | complementation *ano** | this study |
| pBAD-*ano**_start2 | complementation *ano** | this study |
| pBAD-*ano*_start4 | complementation *ano** | this study |
| pBAD-*ano**_start4 | complementation *ano** | this study |
| pBAD-*ano*_start5 | complementation *ano** | this study |
| pBAD-*ano**_start5 | complementation *ano** | this study |

**Supplementary Table S1:** Bacterial strains and plasmids used in this study.
