# Supplementary material for: The Novel Anaerobiosis-Responsive Overlapping Gene ano Is Overlapping Antisense to the Annotated Gene ECs2385 of Escherichia coli O157:H7 Sakai
Source: Front Microbiol. 2018 May 14;9:931. doi: 10.3389/fmicb.2018.00931 (PMC5960689; doi:10.3389/fmicb.2018.00931)
Supplement: TABLE S2 — Oligonucleotides used in this study. Restriction enzyme cut sites are highlighted in bold. [file Table_2.DOCX]

| name | sequence 5’- 3’ | purpose |
| --- | --- | --- |
| ECs2384-*Sal*I-320F | ggct**GTCGAC**taacaccgcatctgttcacg | promotor activity upstream TSS |
| ECs2384-*EcoR*I-40R | gtcg**GAATTC**gttacaagtattacacaaag | promotor activity upstream TSS |
| *ano*+159R | gatcagaagttagtcgataa | 5’RACE, reverse transcription |
| *ano*+81R | cacagccagcaatgcgccttcagt | 5’RACE, 1^st^ PCR |
| *ano*+56R | gagtcagcgcagaatggtgaacca | 5’RACE, 2^nd^ PCR |
| rrsHR | ggaggtgatccaaccgcagg | qRT-PCR 16S rRNA |
| rrsHF | aatgttgggttaagtcccgc | qRT-PCR 16S rRNA |
| *ano*+14F | ctactgcgtcgcgcgtaa | qRT-PCR *ano* |
| *ano*+161R | agacaataaggctgtagatcagaag | qRT-PCR *ano* |
| ECs2384+215R | ttacttgcggtatttagtagcca | qRT-PCR ECs2384 |
| *ano*_S1-*Pst*I-116F | tagt**CTGCAG**gctgctaaagatgacgcagct | EGFP-fusion protein start codon 1 (CTG) |
| *ano*_S2-*Pst*I-107F | atta**CTGCAG**gatgacgcagctcgtgctaac | EGFP-fusion protein start codon 2 (ATG) |
| *ano*_S3-*Pst*I-95F | ttaa**CTGCAG**ggtgctaaccagcgtctggac | EGFP-fusion protein start codon 3 (GTG) |
| *ano*_S5-*Pst*I+1F | ggat**CTGCAG**gctgctatttaccactactgc | EGFP-fusion protein start codon 5 (CTG) |
| *ano*-*Nco*I+167R | ggtc**CCATGG**caagacaataaggctgtagat cag | EGFP-fusion protein |
| ECs2385-*Pst*I+1F | aact**CTGCAG**gatgaaacgcgcgtctttgct | EGFP-fusion protein |
| ECs2385-*Nco*I+983R | aagt**CCATGG**cctgcgtcgcgcgtaacatat | EGFP-fusion protein |
| ECs2384+4F | aagctactaaactggtactgggc | RT-PCR operon ECs2384-*ano;* qRT-PCR ECs2384 |
| pHA5F | cgcaggaaagaacatgtg | amplification pHA1887 |
| pHA3R | aagggcctcgtgatacg | amplification pHA1887 |
| HA3F | aggcgtatcacgaggccctt | amplification mutation cassette |
| HA5R | ctcacatgttctttcctgcg | amplification mutation cassette |
| SM5F | atctcaagagtggcagcggt | amplification selection cassette |
| SM3R | ttatccacctccttgc | amplification selection cassette |
| HA3*ano*-115F | aggcgtatcacgaggcccttgttccgacgttcag gctg | translationally arrested *ano** mutant |
| SM5*ano*mut+19R | accgctgccactcttgagataaccagagcaagg gaatatgttacgcgctacgc | translationally arrested *ano** mutant |
| SM3*ano*mut-5F | gcaaggaggtgcataattttgtctgctatttaccacta ctgcgtagcgc | translationally arrested *ano** mutant |
| HA5*ano*+174R | ctcacatgttctttcctgcgctgtagatcagaagttag | translationally arrested *ano** mutant |
| *ano*-78F | gacaacatggctactaaa | amplification *ano* |
| *ano*+124R | ccggttgcggtgagcagt | amplification *ano;* RT-PCR operon ECs2384-*ano* |
| *ano*_S2-*Nco*I-107F | tatg**ccatgg**gaatgacgcagctcgtgctaac | complementation start codon 2 (ATG) |
| *ano*_S4-*Nco*I-35F | gctg**ccatgg**gagtgaaaaatggcgcacattg | complementation start codon 4 (GTG) |
| *ano*_S5-*Nco*I+1F | gact**ccatgg**gactgctatttaccactactgc | complementation start codon 5 (CTG) |
| *ano*-*Hind*III+170R | tcgt**aagctt**ttaaagacaataaggctgta | complementation |
| pBAD+208F | atgccatagcatttttatcc | amplification pBAD |

**Supplementary Table S2:** Oligonucleotides used in this study. Restriction enzyme cut sites are highlighted in bold.
